# Children’s Learning From Interactive eBooks: Simple Irrelevant Features Are Not Necessarily Worse Than Relevant Ones

**DOI:** 10.3389/fpsyg.2018.02733

**Published:** 2019-01-10

**Authors:** Roxanne A. Etta, Heather L. Kirkorian

**Affiliations:** Human Development and Family Studies, University of Wisconsin-Madison, Madison, WI, United States

**Keywords:** eBooks, books, interactive media, word learning, story comprehension

## Abstract

The purpose of this study was to investigate experimentally the extent to which children’s novel word learning and story comprehension differs for non-interactive eBooks and interactive eBooks with simple relevant or irrelevant interactive features that advance the narrative. An original story with novel word-object pairs was read to preschoolers (3–5 years old, *N* = 103) using one of the three eBook formats: non-interactive control, interactive-relevant, interactive-irrelevant. The book formats differed only in the manner in which the story advanced from one page to the next: children observed the experimenter turn the page (non-interactive), children touched a relevant image on the screen (relevant-interactive), or children touched an irrelevant image on the screen (irrelevant-interactive). Novel word learning and story comprehension were assessed with post-tests in which children picked target objects from an array and sorted story events into their original sequence, respectively. Findings indicate that word learning and story comprehension were similar across all three books, suggesting that simple interactive features – whether relevant or irrelevant to the story – had little impact on preschoolers’ learning in this controlled experiment. Thus, simple interactivity that does not disrupt the story also does not hinder ongoing story comprehension.

## Introduction

Reading aloud to young children supports healthy development (Mol and Bus, 2011). The period from birth to age 5 years is considered an especially critical time for book reading, as it creates a foundation for early language development (Duursma et al., 2008). Reading books aloud to preschool-aged children in particular is associated with a plethora of benefits, including growth in vocabulary (Ard and Beverly, 2004; McLeod and McDade, 2011), language skill (Debaryshe, 1993), emergent literacy (High et al., 2000), and overall academic success (see Bus et al., 1995 for review). Consistent with these findings, the American Academy of Pediatrics recommends that all parents read aloud with their children daily to support early brain development and parent–child relationships (High et al., 2014).

Critically, the vast majority of research on reading aloud to young children has focused on traditional book formats, that is, non-interactive print books. Yet the increasing popularity of mobile screen devices in recent years has increased the potential to access to electronic books, or “eBooks” in the home (Rideout, 2014). While some eBooks contain simple on-screen text and illustrations, others are loaded with interactive features (e.g., hotspots, mini-games, animations). Research has been mixed on the effectiveness of interactive features within eBooks (see Reich et al., 2016 for review). Relatively few studies have directly compared learning from books with or without interactive features, and the relevance of interactive features (i.e., extent to which they are relevant to the ongoing story) has not been investigated systematically using experimentally controlled stimuli in order to isolate causal mechanisms to identify optimal learning conditions.

The purpose of the current study was to fill one gap in the research on learning from interactive eBooks using a carefully controlled experimental design. Specifically, we examined the extent to which simple interactive features that advance the story have an impact on children’s learning from interactive eBooks. We evaluated preschoolers’ word learning and story comprehension from books that varied based on the presence and relevance of simple interactive features that were designed to direct visual attention toward or away from targeted information (i.e., objects that were labeled). This study represents an initial step in examining causal mechanisms underlying children’s learning from interactive eBooks.

### eBooks in the Home: Children’s Access and Parents’ Perceptions

Research has suggested that the number of books in the home, both electronic and print, impacts children’s academic achievement. The presence of as few as 25–50 books has been shown to improve reading test scores by up to one grade level (Evans et al., 2014). This improvement is greatest for families from lower income levels, where each additional book is associated with a measurable increase in reading skill. Historically, socioeconomic status has been linked to children’s access to books (Neuman and Celano, 2001), but the digital divide is closing: nearly all children in the United States between 0 and 8 years of age have access to at least one mobile screen device in their home (Rideout, 2017). Thus, eBooks have the potential to be a boon for lower-income children’s home libraries.

Despite near-universal access to mobile devices in United States homes, many parents report restricting their young children’s access to eBooks, citing a preference for print materials and a belief that print books are better for children’s learning (Rideout, 2014). Although more than half (57%) of parents report reading to their young children a daily basis, children reportedly spend about 26 min per day reading print books and only 3 min per day with eBooks (Rideout, 2017). Rideout also found that lower-income parents are less likely to read to their young children in a typical day. With the pervasiveness of mobile screen devices in homes, eBooks appear to be a missed opportunity as supplemental learning material for young children, particularly for those with limited access to print books. Interactive eBooks could hold promise for these children with limited access to books and adult readers, but eBooks do not yet have established educational merit.

Parents appear to be conflicted over whether and how to use mobile media with their young children. Over 76% of parents believe that children should be spending less time with screen media (Rideout, 2017). However, 69% of parents agree that tablets can be used to support learning and creativity (Marsh et al., 2015). Some parents have claimed to restrict their children’s access to eBooks, and compared to television, computers, and video games, parents have ranked mobile screen devices as the least educational platform (Rideout, 2014). Yet others have looked to eBooks with hopes of supporting young children’s early literacy. Indeed, in an online survey of more than 2000 parents, parents reported that their children use eBooks often and independently: more than one in four parents (29%) reported that their child uses eBooks several times or more each week, and the vast majority (88%) reported that their child sometimes uses eBooks alone (Etta et al., 2018). These conflicting practices and beliefs about eBooks illustrate the confusion that parents face while trying to navigate their children through the “Digital Wild West” (Guernsey et al., 2012).

The seemingly conflicting reports on children’s access to and use of eBooks may be at least partly due to parents’ reasons for choosing to use print versus eBooks with young children. We found that United States parents of preschoolers perceive eBooks and print books as separate entities that serve different purposes (Etta et al., 2018). This study was the first to explore parents’ perceptions on the *purposes* of print books compared to eBooks in relation to children’s actual *use* of these books. Parents in this study perceived print books as catalysts for familial bonding, while they perceived eBooks as tools to entertain and occupy their child. Despite these differences, parents were just as likely to report using print books (28%) and eBooks (23%) for educational reasons. These results suggest that parent opinion separates print books and eBooks as different tools that serve different purposes, but that the two platforms have equal potential for early education. Whether or not parent opinions on the educational value of eBooks are warranted has yet to be established by the research.

### Preschoolers’ Learning From eBooks

While eBooks in the Apple app store boast educational promise, their value for teaching language and literacy skill is often not validated since apps typically do not cite research studies to support their effectiveness (Guernsey et al., 2012). Conversely, extensive research demonstrates that young children can learn from print books. For instance, research has established children’s ability to learn spelling, vocabulary, language, sound, and general knowledge from print books (see Cunningham and Stanovich, 1991; Bus et al., 1995 for reviews).

Just as young children can learn from printed narratives, so too can they learn from narratives presented on screens. This has been studied most thoroughly with television narratives, with decades of research demonstrating that preschoolers readily learn from watching educational television independently (see Anderson and Kirkorian, 2015 for review). More importantly, as with printed books, studies have shown that children learn more from television when they view with a parent who helps them understand what they are seeing (Valkenburg et al., 1999; Barr et al., 2008). Similarly, research has shown that when coviewing a video presentation of a storybook with a parent, preschoolers score higher on story vocabulary and story comprehension when the storybook is interrupted with supportive dialogic reading practices (Strouse et al., 2013). It may be that new mobile screen media offer novel scaffolding affordances through interactive touch screen features, such as hotspots, narration, mini-games, and animations (Bus et al., 2015). Interactive features are prevalent in children’s eBooks, with one study finding that 19 of the top 20 children’s eBooks from the iTunes store contained at least one interactive feature (Guernsey et al., 2012).

Overall, the research on interactive features’ impact on children’s eBook learning has yielded mixed results (see Bus et al., 2015; Reich et al., 2016 for reviews). Some studies suggest that interactivity increases children’s engagement with and learning from books (e.g., Smeets and Bus, 2012, 2014; Estevez-Menendez et al., 2014); but other studies suggest that interactivity disrupts narratives and hinders learning (e.g., de Jong and Bus, 2002; Tare et al., 2010; Chiong et al., 2012; Parish-Morris et al., 2013) or has no effect on learning (e.g., Lauricella et al., 2014).

One reason for these kaleidoscopic findings may be variability in the types of interactive features examined in prior research, which vary both in the complexity of the features and the extent to which they are relevant to the narrative (e.g., simple sound buttons as objects or animals are mentioned in the story versus extensive mini-games that are tangential to the story). In one of the few empirical studies to directly compare different types of eBooks using carefully controlled stimuli, Smeets and Bus (2014) investigated the effects of different types of eBooks on Kindergarteners’ word learning and story comprehension. Children were randomly assigned to either read a static eBook, read an animated eBook, use an interactive-animated eBook, or play an unrelated game (control). Critically, in the interactive-animated eBook, the interactive features were relatively simple and relevant to the story: children could click a hotspot to hear the definition of a target word. While results showed no differences in story comprehension as a function of eBook condition, there were significant condition effects on word learning. Specifically, children performed best on vocabulary learning assessments in the interactive-animated eBook condition and worst in the static eBook condition. Thus, the impact of eBooks on learning may depend not only on the type of interactive features that are present, but also the type of learning that is being assessed.

The interactive features and animations in the experimental eBooks used by Smeets and Bus (2014) were intentionally designed to direct attention to relevant aspects of the lesson, which could explain why they enhanced word learning without compromising story comprehension. Other research suggests that irrelevant features that distract from (rather than support) the story may interfere with learning from eBooks (see Bus et al., 2015 for review). Such findings are consistent with information-processing theories about other types of educational media, which predict that the extent to which children learn from media depends on the extent to which those media reduce cognitive load by using well-integrated narrative and educational content (Fisch, 2000) and simple device mechanics (Strommen, 1993). However, many studies reviewed by Bus et al. (2015) do not use experimentally controlled stimuli and thus confound the relevance of interactive feature with the complexity of those features (e.g., simple hot spots that briefly reveal definitions of target words versus complex, relatively lengthy mini-games that are tangential to the story). Researchers have yet to assess the impact of interactive features in a single study using carefully controlled experimental stimuli that isolate feature relevance while controlling for feature complexity.

### Overview of the Current Study

The aim of the present study was to isolate specific effects of interactive features on young children’s learning from interactive eBooks in order to help identify optimal conditions for learning. Specifically, this study was designed to determine the extent to which learning from eBooks is affected by simple interactive features embedded within the story using experimental stimuli to vary the presence and relevance of those features while controlling for their complexity and duration. Preschoolers (3–5 years; *N* = 103) were randomly assigned to read an eBook that was non-interactive (control) or that contained simple interactive features that were either relevant or irrelevant to learning new words. In order to compare effects of interactivity on different learning outcomes, children completed a post-test for word learning and for story sequencing. Based on prior research and information-processing theories related to children’s learning from educational screen media, we had the following hypotheses:

(1) Word learning. While prior research has found that simple relevant interactive features increase preschoolers’ word learning from eBooks (Smeets and Bus, 2014), information processing theories of educational media posit that irrelevant features increase cognitive load, thus hindering word learning (Strommen, 1993; Fisch, 2000). Therefore, we predicted that a book with relevant interactive features would increase word learning, while a book with irrelevant features would decrease word learning, compared to intermediate learning with a non-interactive eBook.
(2) Story sequence. Prior research demonstrated that even relevant interactive features can interfere with story comprehension as measured by a story sequencing task (Chiong et al., 2012; Parish-Morris et al., 2013; Etta et al., 2017). Information processing theories posit that irrelevant media features will increase cognitive load and therefore disrupt narrative comprehension and learning (Strommen, 1993; Fisch, 2000). Therefore, we predicted that children would be less likely to correctly sequence the story following either interactive eBook than following the non-interactive book, and that performance on this task would be particularly low for children who used an interactive eBook with irrelevant features.

## Materials and Methods

### Participants and Design

The sample included 103 children (58% female) between 3 and 5 years of age (*M*_age_ = 4.27, range = 3.00–5.95). Recruitment occurred through voluntary participation at a children’s museum in a university town in the upper Midwest United States. Approval from the Institutional Research Board for Education and Social/Behavioral Science at the University of Wisconsin-Madison was acquired prior to recruitment, parents provided written informed consent for their child to participate, and children provided verbal assent. In a between-subjects design, children were randomly assigned to one of three groups: non-interactive (*N* = 37), relevant-interactive (*N* = 30), or irrelevant-interactive (*N* = 36).

Of the 79 parents (76% of the sample) who responded to the parent survey, the majority (82%) identified their child’s race and ethnicity as white and non-Hispanic. Parent education averaged 17.12 years (range: 12–25), which is equivalent to slightly more than a 4-year college degree. Parents also completed the MacArthur Scale of Subjective Social Status by placing themselves on a ladder representing a range between people who have the least money, little or no education, and no job or a job that is not respected (1) and people who have the most money, highest level of education, and highly respected jobs (10) (Goodman et al., 2001). Parents’ average status on this measure was 7.85 (range: 5 to 10). Parents reported owning an average of 3.56 mobile screen devices upon which eBooks can be read. In concordance with the findings from Rideout (2017), parents reported that children read print more often than electronic books. The vast majority (89%) of parents reported that their child reads traditional print books daily, while interactive eBooks were used less frequently (90% reported “occasionally” or “almost never”).

### Book Stimuli

In order to provide experimental control over the stimuli and eliminate familiarity effects, novel eBooks were written and illustrated by the first author. In the story, a farmer needs to collect his tools to fix a broken tractor. These “tools” were five novel objects with corresponding novel labels from the Novel Object and Unusual Name Database, 2^nd^ Edition (NOUN database; Horst and Hout, unpublished). These novel word-object pairs from the NOUN database are used extensively in research with young children, have similar phonetic properties to common English-language nouns, and ensure that participants would not have any prior knowledge of the target words presented in the book. As the story progressed, children found the hidden novel object-label pairs one by one. The story was designed to be engaging but simple, as to not overburden children’s capacity for learning from a single exposure (Rice et al., 1982).

**FIGURE 1:**
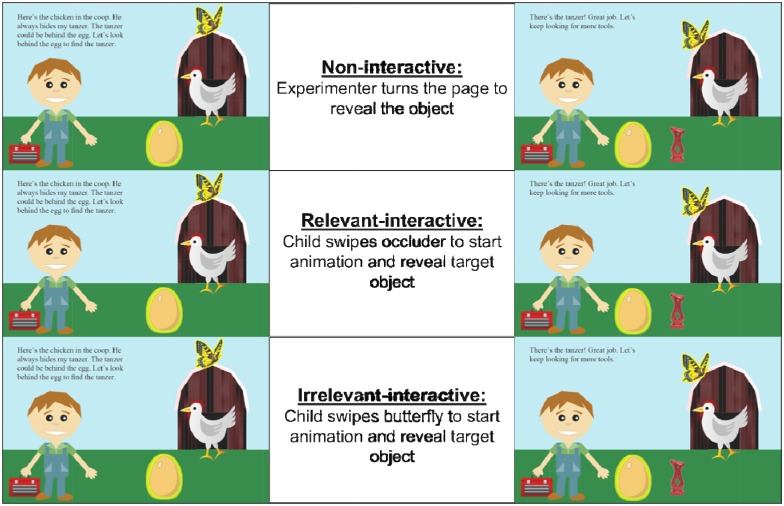
Sample pages from the eBook. The page on the left depicts a scene before the target object is revealed. The page on the right depicts a scene after the experimenter turns the page (non-interactive condition), the child swipes the occluder to the side to reveal the target object (relevant condition), or the child swipes the butterfly, launching an animation of the butterfly moving around the screen while the occluder moves to the side (irrelevant condition).

eBooks were presented on a Samsung Galaxy Tab 10.1 with a 25.7 cm display (1280 × 800 pixels). See Figure 1 for sample pages. The three versions of the book were identical except for the manner in which each of the five novel objects was revealed. In the non-interactive book, the novel object was revealed when the experimenter turned the page (i.e., swiped the screen). In the interactive books, children were instructed to reveal the novel object by activating the manipulative feature: in the relevant-interactive book, participants activated the manipulative feature by swiping left on the object that occluded the tool, launching an animation of the occluder moving to the side and revealing the novel target object. This condition was intended to direct visual attention to the target object with a moderate-salience animation as the target object was revealed. In the irrelevant-interactive book, participants swiped left on a butterfly to make it flutter across the screen; simultaneously in the background, the occluding object moved to the side. This condition was intended to direct visual attention away from the target object with a high-salience animation as the target object was revealed. The story content in all books were identical across conditions.

For all versions of the eBook, both manipulative features (i.e., irrelevant butterfly and relevant occluder covering the novel object) were highlighted with a bright yellow outline as a visual cue, so that the only visual difference between the three conditions was the animation (or lack thereof) that occurred as each target object was revealed. The text was also identical for each version of the eBook. Each novel object was labeled three times while it was occluded, and once more after the novel object was revealed.

### Procedure

Children were tested individually in a private room at a local children’s museum. An experimenter interacted with children while an observer recorded children’s responses. Children provided assent before the study began. Upon their arrival, children were asked, “I have some books to read and games to play; would you like to read and play with me?” Children were also reassured there would be no negative consequences if they wished to prematurely withdraw from the study. Prior to book reading, the experimenter stated, “If you want to stop reading or playing at any time, you can let us know. You will not get in any trouble if you want to stop.” Once children provided verbal assent, the experimenter read the story aloud once to the child. After book reading, children completed tasks to assess word learning, story comprehension, general story schema, and receptive vocabulary. At the end of the study, children received a small gift for participating. The entire session lasted approximately 15 min.

#### Word Learning

**FIGURE 2:**
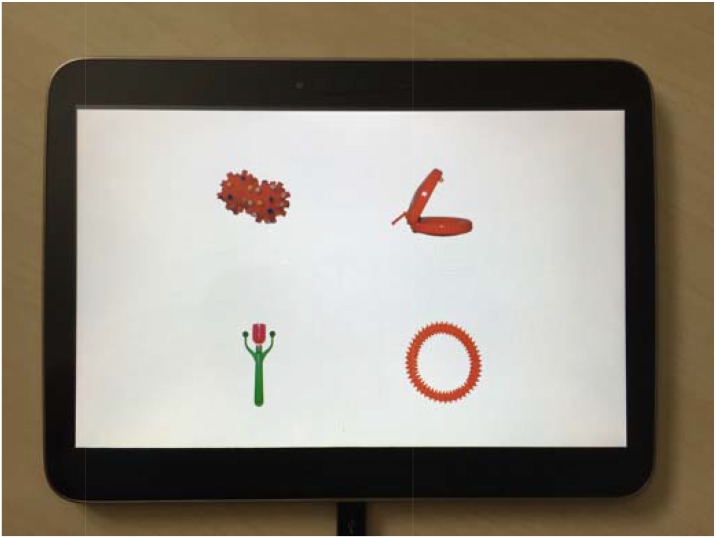
Example of the word learning task presented at the end of the eBook.

The word learning assessment was presented on the last five pages of the eBook. All of the five novel object-label pairs were tested once in a randomized order. Testing pages were similar to those from fast-mapping word learning research in which young children demonstrate a robust ability to learn words for novel objects after a single exposure (e.g., Vlach and Sandhofer, 2011). Each testing page contained an array of four objects from which the participant could choose. For each testing page, the experimenter asked participants, “Which one of these is the *[novel label]*? Can you point to the *[novel label]*?” The array on each testing page included the target object (i.e., correct novel object from the story), a familiar distractor (i.e., another novel object that was featured in the book but had a different label), a perceptually similar distractor (i.e., object from the NOUN database that was not in the story but was the same color as the target object), and a perceptually different distractor (i.e., object from the NOUN database that was not in the story and that was a different color). The observer recorded which of the four objects children selected through pointing. The subsequent dependent variable for word learning was the total number of novel objects correctly identified (range: 0–5), with chance at 1.25 (i.e., 0.25 ^∗^ 5 trials). See Figure 2.

#### Story Comprehension

**FIGURE 3:**
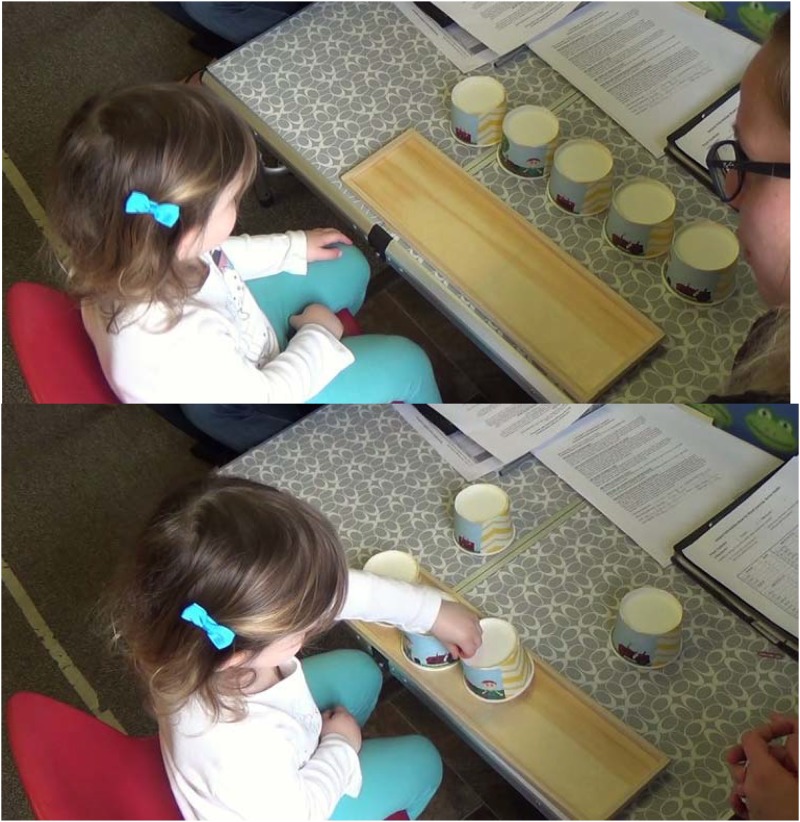
Story comprehension task with shuffled cups **(top)** and sorting execution **(bottom)**.

Children completed a story comprehension task that was adapted from the Parish-Morris et al. (2013) story comprehension test. Five pictures of events from the story were printed and attached to cups (see Figure 3). These “game pieces” represented integral story content to represent the simple story arc: Farmer Fred learns that his tractor is broken, Fred’s toolbox is empty, Fred finds his tools, Fred fills his toolbox, Fred fixes his tractor. The game pieces were presented out of order, and children were asked to place the cups on the game board in their correct sequence. First, the experimenter gave a brief description of the event occurring in each of the five pictures. Then, the experimenter asked questions like, “Which one came first? Can you put it here?” The observer recorded the order in which children placed the cups on the game board. Subsequently, the experimenter assigned a story comprehension score using Kendall’s tau (Kendall, 1938), which is a type of correlation coefficient that is used for ordinal (as opposed to continuous) variables. Specifically, Kendall’s tau is a rank correlation coefficient that is calculated by comparing the ordinal observations of two groups, in this case the correct event sequence and the sequence produced by a child. Kendal’s tau can be interpreted in the same way as other correlation coefficients with a range from -1.0 (perfect inverse correlation) to 0 (no correlation) to 1.0 (perfect positive correlation). When ordinal observations of two groups are similar (i.e., if participants place the cups in the correct order), scores are positive and closer to 1 than to 0. When ordinal observations are dissimilar (e.g., if participants place cups in a random sequence), tau scores are closer to zero or even below zero. Thus, the possible range of scores was -1 to 1, with zero indicating no association and 1 indicating a perfect sequence.

#### General Story Schema

After completing the story comprehension task, children played another seriation game using analogous materials from a similar (but unfamiliar) story in order to assess general story schema and understanding of the task itself. This task was designed to measure children’s prior story schema by assessing how well-children could identify the beginning, middle, and end of a story that they have never heard. Again, the game pieces represented integral elements of a story arc, but from an unfamiliar story. In this story, Zookeeper Zach learns that his car is broken, Zach’s basket is empty, Zach finds his tools, Zach fills his basket, Zach fixes his car. The procedure was the same as the story comprehension task: the experimenter briefly described what happened in each picture and then asked the children to choose which picture came first, second, and so on. The general story schema task was also scored by calculating Kendall’s tau coefficient, comparing each child’s sequence with the correct sequence.

#### Vocabulary

**FIGURE 4:**
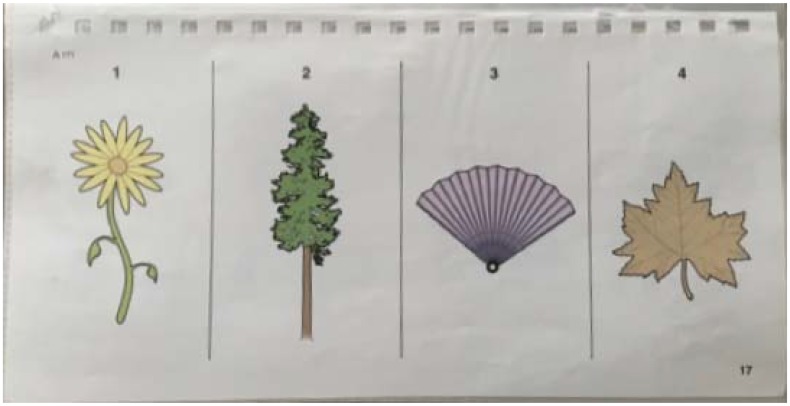
Example page of the Receptive One Word Picture Vocabulary Test – Fourth Edition (Martin and Brownell, 2010).

Receptive vocabulary was assessed using the Receptive One Word Picture Vocabulary Test – Fourth Edition (Martin and Brownell, 2010). Six participants did not complete the vocabulary assessment, so their scores were replaced with the group mean. The pattern of results was unchanged when these children were removed from analyses. For each word, children were shown four pictures and asked to point to the picture that matched a word (e.g., “Flower. Which one is flower?”). See Figure 4. As the task advanced, the words became more difficult. The test began within the section corresponding to the child’s age and ceased when the child answered six out of eight consecutive questions incorrectly. Throughout the assessment, the research assistant coded performance on each word and then calculated a raw score by subtracting the total number of errors from the total number of words tested. After the session ended, raw scores were converted to standardized scores from the assessment manual based on the child’s age. The population distribution of standardized scores has a mean of 100 and a standard deviation of 15.

### Parent Survey

In order to provide descriptive information about the study sample, parents of the participants were asked to complete a short online survey that contained demographic questions (e.g., race, ethnicity, parent education, subjective social status) and their child’s exposure to different book formats and media. Parents either completed the survey while their child was participating in the study or were emailed a survey link after their child participated. Of particular interest was parent-reported use of print and eBooks at home. Parents were asked to rate how frequently their children use eBooks and print books (e.g., never, occasionally, sometimes, daily).

## Results

### Analytic Approach

The current study was designed to assess the impact of eBook interactivity on children’s learning. Separate analyses of covariance (ANCOVA) were used to test the effect of interactivity (non-interactive, relevant-interactive, irrelevant-interactive) on two dependent variables: word learning and story comprehension. See Table 1 for descriptive statistics for and correlations between the dependent variables and potential covariates. Preliminary analyses indicated that child age was correlated with both dependent variables, and that receptive vocabulary and general story schema were correlated with story comprehension even after controlling for age (see Table 1). Thus, both ANCOVAs included age as a covariate, and the story comprehension analysis also included vocabulary and general story schema as covariates. Results of the ANCOVAs for word learning and story comprehension can be found in Tables 2, 3, respectively.

**Table 1:** Descriptive statistics, zero-order correlations, and partial correlations controlling for age.

	Descriptives		Correlations				
	Mean (*SD*)	Range	1	2	3	4	5
(1) Child age (years)	4.27 (0*.77*)	3.00–5.95	—	—	—	—	—
(2) Vocabulary	108.34 (*11.83*)	66–142	-0.07	—	0.28**	-0.11	0.37**
(3) General story schema	0.19 (*0.43*)	-0.6–1.0	0.50**	0.21*	—	-0.15	0.38**
(4) Word learning	2.65 (*1.28*)	0–5	0.23*	-0.12	0.13	—	-0.11
(5) Story comprehension	0.31 (*0.46*)	0.4–1.0	0.37**	0.32*	0.48**	-0.01	—


*Numbers below the diagonal are zero-order bivariate correlations while the numbers above the diagonal are first-order partial correlations controlling for age. N = 103. ^∗^p < 0.05, ^∗∗^p < 0.01.*

**Table 2:** Final ANCOVA for novel word learning.

	MS	*F*
Intercept	3.00	1.90
Child age	9.39	5.92^∗^
Condition	0.60	0.37


*The dependent variable was the number of novel objects correctly identified (out of 5). N = 103. ^∗^p < 0.05.*

**Table 3:** Final ANCOVA for story comprehension.

	MS	*F*
Intercept	1.14	7.74^∗∗^
Child age	0.67	4.60^∗^
Vocabulary	1.17	7.99^∗∗^
General story schema	1.61	10.94^∗∗^
Condition	0.15	1.03


*The dependent variable was the story comprehension task score (ranging from -1 to 1). N = 103. ^∗^p < 0.05, ^∗∗^p < 0.01.*

Preliminary analyses indicated that child sex, child race, parent-reported subjective social status, and parent-reported book use (both print and electronic) did not differ significantly across experimental conditions. Moreover, these characteristics did not predict performance on any of the outcome measures, so they were not considered further.

### Word Learning

**FIGURE 5:**
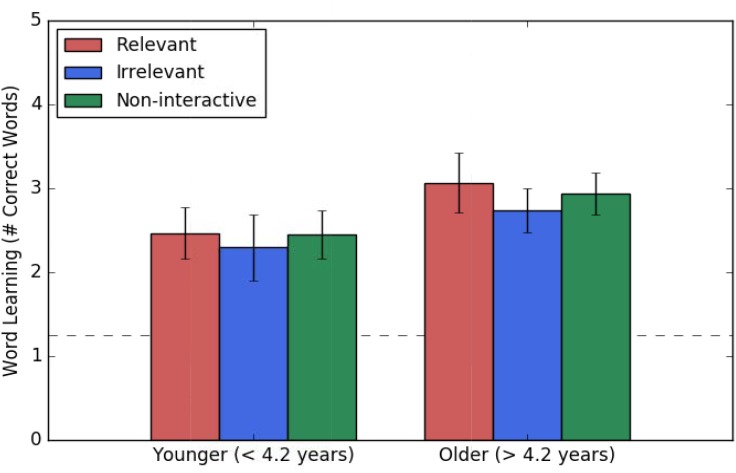
Mean number of correct responses for the word-learning test as a function of age group and condition. Age was a continuous predictor in analyses but is depicted here as two median-split groups for illustration only. The y axis represents the possible range of scores from 0 to 5. The horizontal dashed line represents chance at a score of 1.25. Bars represent +/– one standard error above/below the mean.

Word learning did not differ by interactivity, *F*(1,99) = 0.37, *p* = 0.689. Children learned significantly more words than chance *t*(102) = 11.09, *p* < 0.001. There was an age effect such that older children performed better than younger children on word learning *F*(1,99) = 5.92, *p* = 0.017. The results of the ANCOVA for story comprehension can be found in Table 2. Word learning scores are plotted in Figure 5 as a function of age and interactivity.

Performance on each individual word indicated a primacy effect (Murdock, 1962) such children correctly identified the first (76% correct) and second (67%) objects more often than the last three (28, 46, 48%). Importantly, this effect did not vary by condition for the first and second objects χ^2^(2, *N* = 103) = 1.21, *p* > 0.05; χ^2^(2, *N* = 103) = 0.29, *p* > 0.05. Exploratory investigation of children’s errors on this task revealed that if children selected an incorrect target object from the array, 73% of the time it was a familiar (but incorrect) object from the story. A *post hoc* analysis indicated that the rate of this type of error did not differ significantly by condition, *F*(1,90) = 1.36, *p* = 0.261.

### Story Comprehension

**FIGURE 6:**
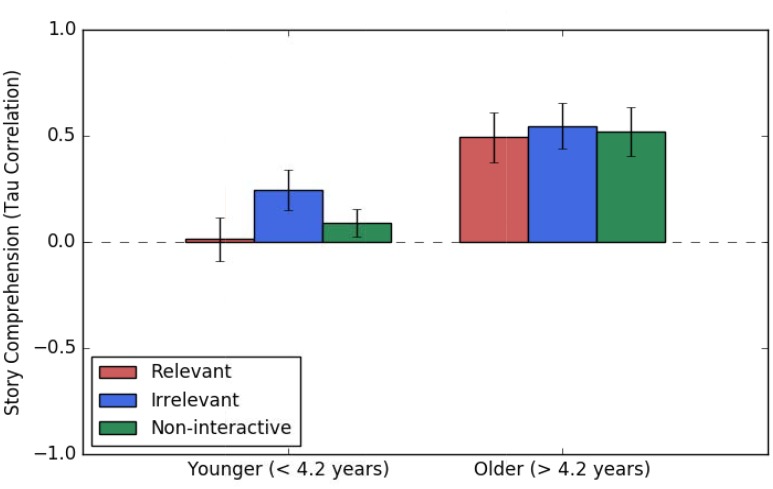
Mean story comprehension as a function of age and condition. Age was a continuous predictor in analyses but is depicted here as two median-split groups for illustration only. Story comprehension was operationalized as a tau correlation between the story sequence produced by the child and the correct story sequence. The y axis represents the possible range of scores from –1 to 1. The horizontal dashed line represents a correlation of zero (i.e., no systematic association between the sequence produced by the child and the correct sequence). Bars represent +/– one standard error above/below the mean.

As with word learning, children’s story comprehension scores did not differ based on interactivity *F*(1,97) = 1.03, *p* = 0.362. There was an age effect such that older children performed better than younger children on story comprehension, *F*(1,97) = 4.60, *p* = 0.034. Story comprehension scores also increased as a function of general story schema and standardized vocabulary, *F*(1,97) = 10.94, *p* = 0.001, and *F*(1,97) = 7.99, *p* = 0.006, respectively. The results of the ANCOVA for story comprehension can be found in Table 3. Story comprehension scores are plotted in Figure 6 as a function of age and interactivity.

## Discussion

Interactive features are prevalent in children’s eBooks. A content analysis found that 19 of the 20 top children’s eBooks from the iTunes store had at least one interactive feature (Guernsey et al., 2012). Despite the prevalence of interactive features in popular eBooks for young children, parent perceptions and research findings have been mixed regarding the impact of interactivity on children’s learning from eBooks. Such mixed findings may be partly due to differences in the type of learning that is assessed and in the relevance and complexity of the interactive features. This study was designed to specifically investigate the effect of simple interactive features that were either relevant or irrelevant to the story on preschoolers’ word learning and story comprehension. In order to maintain high experimental control, the interactive features in the relevant and irrelevant conditions were simple and similar in nature (i.e., hot spots that launched a short animation). These features were designed to direct visual attention toward (relevant) or away from (irrelevant) target objects. Contrary to predictions, these simple interactive features did not appear to affect learning.

### Word Learning

Information processing theories posit that the extent to which young children learn from educational media will depend partly on the extent to which the story content and interactive features increase cognitive load (Strommen, 1993; Fisch, 2000). Researchers such as Bus et al. (2015) agree that “hypermedia” features, such as irrelevant interactivity, increase cognitive load and lead to poorer learning outcomes for children. Previous research suggests that simple, relevant hotspots can increase learning from eBooks (Smeets and Bus, 2014) while complex, irrelevant features such as mini-games disrupt word learning (Takacs et al., 2015). In the case of print books, even simple irrelevant interactivity (i.e., lifting a flap to reveal the target object) can be harmful for children’s word learning (Tare et al., 2010). However, in the current study we found no evidence that simple relevant features increase word learning or that simple irrelevant features disrupt word learning, compared to a non-interactive eBook.

Our irrelevant feature was intended to direct attention away from target objects. However, the scripted timing of these features may have ameliorated any potential negative effect on word learning. While some eBooks have interactive hotspots that children can activate at any point in the story (e.g., Smeets and Bus, 2014), in our book children could only activate this feature during scripted break points in the story (i.e., between pages). Perhaps interactive features – at least simple, short ones – have minimal effect at these scripted breaks.

Conversely, our relevant interactive feature may have had little benefit for word learning because it did not introduce any new information that would help children learn the word. Our relevant features only served to direct visual attention to the target objects as they were revealed. We may have seen a more obvious effect of relevant interactivity if the relevant features added new content, such as repeating or defining each word (Smeets and Bus, 2014).

It is also important to note that in order to ensure word learning from a single exposure, the story itself was fairly simple: there were few visual distractors on the page (other than the ones we put there intentionally), There was a predictable format each time a new target object was found, the novel label was repeated several times per object, and the label was provided both before and after the object was revealed. It may be that simple, irrelevant features have little impact when there is so much other support for word learning. Future research could test the extent to which the structure, predictability, and timing of interactive features have an impact on learning from eBooks.

Although the book was simple and contained many supports for word learning (e.g., repetition before and after the object was revealed), the average word learning score was far from perfect, with an average score of about 2.6 out of 5. This average is better than chance (1.25), but it is lower than expected given that young children can fast-map new words to objects after a single exposure (e.g., Vlach and Sandhofer, 2011). It is possible that the butterfly, which appeared on each page in all three conditions, and whose location changed from one page to the next, created a visual distraction in all conditions (regardless of interactivity). Future research could test systematically the extent to which word learning from eBooks is affected by the amount and complexity of visual distractors on the page. Additionally, future research could use alternative assessments for word learning that focus on depth of word knowledge (e.g., extension to other exemplars, delayed recall) to further examine whether interactivity has an impact on word learning from eBooks (Hoffman et al., 2014).

It is also noteworthy that the vast majority of children’s errors on the word learning test were for the familiar distractor; that is, when children answered incorrectly, it was usually because they selected another object that was in the book but that had a different label. The erroneous selection of familiar objects from the story suggests that although children could not always remember the exact object-label pairing from the book, they were able to recognize which objects were from the story. Put differently, the likelihood that children selected an object from the book was far greater than chance, but the likelihood that they then distinguished between those objects based on their novel label was not. Nonetheless, the likelihood that children chose the familiar distractor did not differ by condition, and so was not affected by interactivity. Perhaps with multiple book readings, differences would arise in children’s ability to pair a label with its correct object based on interactivity level.

### Story Comprehension

Contrary to previous research (de Jong and Bus, 2002; Chiong et al., 2012; Etta et al., 2017), children in the interactive book conditions performed as well as those in the interactive book condition on story comprehension. One previous study (Lauricella et al., 2014) had similar findings insofar as children in this study attended to interactive books more than non-interactive books, yet story comprehension was equivalent for these books. Again, perhaps the interactive features were well-enough embedded into the story to ameliorate any negative impact of interactivity on story comprehension, particularly given that children were only allowed to use the interactive features during scripted pauses in the story.

As noted previously, the goal of the current study was not to test the impact of maximally distracting eBook features. Rather, the goal was to isolate one factor that could affect learning from eBooks (i.e., presence and relevance of simple interactivity) while holding constant other potentially important factors (e.g., complexity, duration, and timing of interactive features). In doing so, we found that interactive features – whether relevant or irrelevant to the story – had little impact on story comprehension when those interactive features were simple animations that advanced the story. Future studies could adopt a similar experimental approach to isolate other factors and examine eBooks that incorporate more elaborate disruptive features with visual, auditory, and haptic cues, such as mini-games, to distract the reader.

### Limitations and Future Directions

One important limitation of the current study that should be addressed in future research is the homogeneity of this convenience sample: children were predominantly white with highly educated, well-off parents who reported reading print books to their children daily and limiting screen time. It remains to be seen whether these findings would be replicated in children who have other demographic characteristics, have less access to print books and lower existing story schema, or are more likely to engage in shared screen time with their parents. Furthermore, in order to isolate causal mechanisms, an unfamiliar experimenter read the eBooks to children using a well-rehearsed script. Future research should aim to enhance generalization by including parents in this type of research. Indeed, previous research has found that children learn more content from eBooks when reading with a parent compared to hearing a simple audio-recorded narration (Dore et al., 2018), but the quality of parent–child dialog may be influenced by interactive features (Chiong et al., 2012).

## Conclusion

The current study was designed to isolate and examine the effects of simple interactive features that varied in their relevance to a simple story. Findings indicated that relevant attention-directing interactive features were no better than irrelevant attention-directing features in supporting word learning or story comprehension. These results suggest that simple, repetitive interactive features that do not remove the reader from the story are not necessarily helpful nor harmful for learning. This study adds to the growing body of mixed results regarding young children’s learning from eBooks, emphasizing the complexity and diversity of this particular medium. Our findings suggest that simple interactive features that advance the story do not exceed preschoolers’ capacity to comprehend and learn from the story.

## Ethics Statement

This study was carried out in accordance with the recommendations of Education and Social/Behavioral Science Institutional Review Board. The protocol was approved by the Institutional Review Board. All subjects gave written informed consent in accordance with the Declaration of Helsinki.

## Author Contributions

RE was primarily responsible for collecting and analyzing data and also significantly contributed the manuscript write-up and assisted with study design. HK was primarily responsible for creating the study design and seeking funding from the Spencer Foundation and oversaw data analysis and write-up, providing detailed feedback throughout.

## Conflict of Interest Statement

The authors declare that the research was conducted in the absence of any commercial or financial relationships that could be construed as a potential conflict of interest.
